# The Differentiation in Image Post-processing and 3D Reconstruction During Evaluation of Carotid Plaques From MR and CT Data Sources

**DOI:** 10.3389/fphys.2021.645438

**Published:** 2021-04-16

**Authors:** Fengbin Deng, Changping Mu, Ling Yang, Rongqi Yi, Min Gu, Kang Li

**Affiliations:** ^1^North Sichuan Medical College, Nanchong, China; ^2^Department of Radiology, Chongqing General Hospital, University of Chinese Academy of Sciences, Chongqing, China

**Keywords:** carotid plaque, fluid-solid interaction, aneurysms, computational fluid dynamic, CT angiography, MR angiography

## Abstract

**Background:** Carotid plaque morphology and tissue composition help assess risk stratification of stroke events. Many post-processing image techniques based on CT and MR images have been widely used in related research, such as image segmentation, 3D reconstruction, and computer fluid dynamics. However, the criteria for the 3D numerical model of carotid plaque established by CT and MR angiographic image data remain open to questioning.

**Method:** We accurately duplicated the geometry and simulated it using computer software to make a 3D numerical model. The initial images were obtained by CTA and TOF-MRA. MIMICS (Materialize’s interactive medical image control system) software was used to process the images to generate three-dimensional solid models of blood vessels and plaques. The subsequent output was exported to the ANSYS software to generate finite element simulation results for the further hemodynamic study.

**Results:** The 3D models of carotid plaque of TOF-MRA and CTA were simulated by using computer software. CTA has a high-density resolution for carotid plaque, the boundary of the CTA image is obvious, and the main component of which is a calcified tissue. However, the density resolution of TOF-MRA for the carotid plaque and carotid artery was not as good as that of CTA. The results show that there is a large deviation between the TOF-MRA and CTA 3D model of plaque in the carotid artery due to the unclear recognition of plaque boundary during 3D reconstruction, and this can further affect the simulation results of hemodynamics.

**Conclusion:** In this study, two-dimensional images and three-dimensional models of carotid plaques obtained by two angiographic techniques were compared. The potential of these two imaging methods in clinical diagnosis and fluid dynamics of carotid plaque was evaluated, and the selectivity of image post-processing analysis to original medical image acquisition was revealed.

## Introduction

Stroke is the most frequent neurological disease worldwide and one of the leading medical burdens, and it is one that will keep increasing over the next few decades (Seshadri et al., 2006; Lozano et al., 2012; Go et al., 2013). Ischemic cerebrovascular disease accounts for the largest proportion of strokes (Lozano et al., 2012). Ischemic cerebrovascular disease is a multi-factor disease in which atherosclerosis is the most important factor (Lozano et al., 2012). The most obvious form of atherosclerosis is plaque, and the most common cause of cerebrovascular events is carotid atherosclerotic plaque (Go et al., 2013).

As we know, the carotid atherosclerotic plaques may play an important role in the development of ipsilateral ischemic events (Gupta et al., 2015a). In the study of carotid plaque, it is important to consider that the contribution of different features to the occurrence of cerebrovascular events varies greatly. Some morphological, biomechanical, and pathological features are thought to be related to plaque progression (Takaya et al., 2006; Chrencik et al., 2019; Wang et al., 2019). First of all, morphological and biomechanical features such as plaque area and plaque burden can be used to predict the progression of plaque and have been proved more accurate than a single risk factor (Wang et al., 2019). In addition, little change in plaque geometry and quantitative analysis of pathological tissue composition is useful to track plaque progression or regression and the assessment of the risk of plaque rupture (Sheahan et al., 2018; Chrencik et al., 2019; Lu et al., 2019). More recently, studies have suggested that plaque progression may also be related largely to hemodynamics (Eshtehardi et al., 2017).

In order to obtain the corresponding plaque data, clinical high-precision instruments are used to scan plaques. At present, there are many clinical methods for carotid plaque examination, including transcranial Doppler ultrasound (TCD), CT angiography, high-resolution MR, MR angiography, digital subtraction angiography (DSA), etc (Saba et al., 2008; van’t Klooster et al., 2012; Etesami et al., 2013; Nieuwstadt et al., 2015). These methods can provide information about the degree of artery stenosis and plaque characteristics, such as plaque geometry, composition, and stability. CFD is being explored by more and more scholars as an auxiliary tool to study the hemodynamics of patients, especially study the hemodynamics of patients with an asymptomatic carotid plaque for the prevention of stroke (Tang et al., 2013; Jia et al., 2017). 3D image data in any DICOM format can be used as a source of raw information, and any commercial CFD software can generate a corresponding 3D model. However, these detection data are usually only used to exploring the internal characteristics of carotid plaque at present, with little attention paid to the influence of plaque morphology and volume on the development of cerebrovascular diseases. It seems feasible to establish a theoretical model of carotid artery plaque based on computer and to study its possible geometric shape to explore the external features of the plaque to provide a more intuitive method for clinical workers to assess the risk stratification of stroke.

Until recently, most computer modeling studies have focused on specific patient carotid plaque and associated the mechanical stability of specific carotid plaques with clinical events. With the rapid development of computer software, people are more and more aware of the important role of the establishment of automatic recognition, plaque morphology, biomechanics, and 3D pathology model in predicting plaque progress. However, the 3D modeling standards based on CTA and TOF-MRA images are not uniform, especially after image post-processing, the influence of different image types on 3D models and computer mechanical simulation has not been clarified. Our study chose CTA and TOF-MRA examination techniques that are more widely used in clinical practice and explored their clinical application potential. We assessed the potential of CTA and TOF-MRA images to reconstruct a three-dimensional model of carotid plaque and provided a reference for clinicians to assess risk stratification of cerebrovascular events.

## Materials and Methods

### Ethics

1. Ethics committee approval: The studies involving human participants were reviewed and approved by the Ethics Committee of Chongqing People’s Hospital.
2. Consent procedures: Written informed consent to participate in this study was provided by the participants.

### Investigated Subjects

Written informed consent was obtained from the individuals for the publication of any potentially identifiable images or data included in this article. The experimental data collected from a 94-year-old woman with ischemic cerebrovascular disease in Chongqing General Hospital. The patient underwent advanced carotid CTA examination and then TOF-MRA examination for further diagnosis.

### Examination Methods

#### CTA Examination of Carotid

CTA was done by using a helical CT scanner (Philips/Brilliance 64, KVP 120 kV) with multiple lines of detection. A routine cervical cross-sectional CT scan was performed on the patient first, ranging from aortic arch level to skull base level, followed by a plain scan sequence. The size of the fault images was 512/512 px, the pixel size was 0.488281 mm, the field of view was 250.00 mm, the number of images was 340, the thickness of the fault was 0.8 mm, the increment was 0.4 mm.

#### TOF-MRA Examination of Carotid

TOF-MRA was done by using a 1.5 T magnetic resonance scanner (SIEMENS/Avanto) with standard quadrature neck array coils. The size of the fault images was 288*384 px, the pixel size is 0.52083 mm, the field of view is 150.00 mm, the number of fault layers is 94, the thickness of the layer is 0.9 mm, and the increment is 0.9 mm.

### Model Segmentation and Reconstruction

Firstly, the region segmentation of the carotid artery and plaque can be achieved by the gray threshold segmentation method (Moussallem et al., 2012) in which the CTA and MR thresholds of the internal carotid artery are 50–1,400 hu and 80–343, respectively, and the CTA and MR thresholds of plaque are 300,1,350 hu and 100–267, respectively. Secondly, dynamic region growth (Stensjøen et al., 2015) was used to reconstruct carotid artery and plaque surface models in mimics. The model vulnerability could be further repaired and properly optimized by tailoring redundant branches and noise in the 3-Matic assembly where the internal carotid wall thickness was 0.45 mm (the average diameter of the carotid model in this study is about *d* = 4.5 mm, and the wall thickness is generally 8–10% of the vascular diameter). Finally, the grid of the model was subdivided to meet the requirements of mesh accuracy, and the carotid flow cavity model in step format was derived.

### Finite Element Simulation

The fluid properties and boundary conditions were as follows: blood density in internal carotid $\rho =1050\;kg/m^{3}$, blood viscosity $\mu =3.5\;g\cdot {\left(m\cdot s\right)}^{-1}$, and average velocity in the internal carotid artery v=0.18m / s. The vascular wall properties and boundary conditions were as follows: density 2 g/cm^3^, elastic modulus 2.7 Mpa, and Poisson’s ratio 0.45 (approximate incompressible material). Finally, the simulation of carotid blood flow was completed in the ANSYS Fluent module, and the discrete scheme of differential equation group for flow field calculation adopts the upwind accurate upwind scheme, the maximum root means square residual is set at 10^−4^, and the time step for flow field and solid calculation was 10 ms.

## Results

### Comparative Analysis of Image Characteristics of CTA and MR

**Figure 1:**
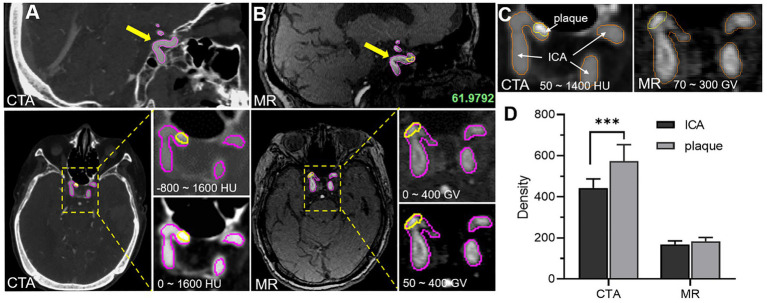
Comparative analysis of image characteristics of CTA and TOF-MRA **(A)** CTA images of carotid artery and plaque, and yellow and magenta circle showed the plaque area and carotid artery area separately **(B)** MR images of carotid artery and plaque. **(C)** The density of carotid artery and plaque density by the two methods. **(D)** The statistical difference of carotid artery and plaque density between the two methods.

In order to visually compare the imaging differences of the carotid artery and plaque between CTA and TOF-MRA, the Mimics software was used to segment the carotid artery and plaque, and the relative density (gray value) of the corresponding tissue was further counted. In this work, the tomographic images of carotid artery and plaque got from CTA and TOF-MRA showed the difference in a grayscale of the carotid artery and plaque from the sagittal plane (sagittal plane) and cross-section (transverse plane), the partial enlarged images revealed the significance of different gray thresholds for segmenting carotid artery and plaque (Figures 1A,B). At the appropriate gray threshold (CTA was 50–1400HU and MR was 70–300GV), it could be better distinguished from surrounding tissues (Figure 1C). Among them, the density of CTA plaque and carotid artery were quite different, which made it easy to distinguish the tissue boundary. The density means, the variance of the carotid, and the variance of carotid arteries and plaques in multiple slices were further counted (Figure 1D). The statistical results showed that, when ignoring the dimensional density difference of the two imaging methods, CTA imaging could better show the difference between carotid artery and plaque through the grayscale of the image compared with TOF-MRA. In addition, since the main component of the plaque in this study was calcified plaque (density range was 300–800HU), only a small amount of components were fibrotic and lipid plaque, this statistical result showed that the CTA is better than TOF-MRA in detecting carotid calcified plaque.

### 3D Reconstruction of CTA and MR Images

**Figure 2:**
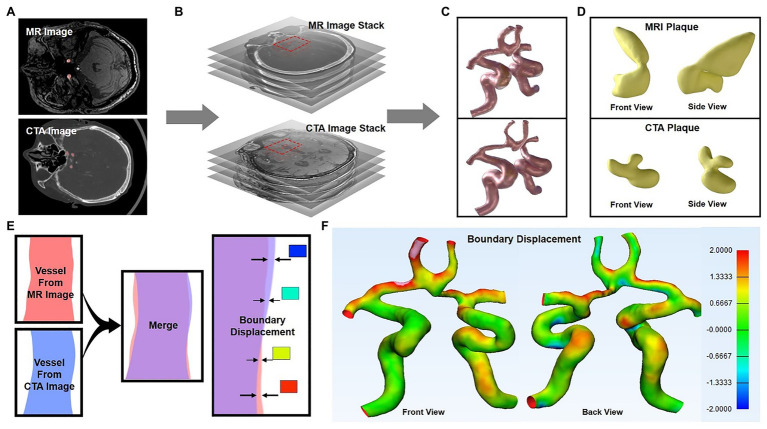
3D reconstruction of CTA and TOF-MRA images **(A–C)** show the 3D reconstruction process of the layer scan image in MIMICs software **(C)** 3D images of blood vessel reconstruction (upper: TOF-MRA, lower: CTA), and the yellow area was the plaque in the blood vessel **(D)** The segmented plaque, front view, and side view show the shape of the plaque from two directions **(E)** Schematic diagram: two 3D models were superimposed, and the position difference of their outer surfaces were showed in different colors **(F)** The position difference of the two models was displayed on the model in the form of a heat map.

The 3D reconstruction model based on CTA and TOF-MRA layer scan images is of great significance for intuitive understanding and judgment of physiological and pathological structures. However, due to different imaging principles, the gray image distribution characteristics are different, and the physiological structure boundaries of the image segmentation would also be different, which further affects the appearance of the final 3D image. In this work, 3D reconstruction of the CTA and TOF-MRA images of the patient at the same lesion (Figures 2A,B) was carried out, and the morphological and structural features were compared. The results showed that the shape of the blood vessel was basically the same, but the thickness of multiple sites was significantly different (Figure 2C). The difference in the position of the boundary showed these areas of significant change in the heat map (Figures 2E,F). In addition, the shape and quality of the plaque as shown in the figure (Figure 2D) were also different, and its position in the blood vessel had even been shifted. The faint differences of 2D images between blood vessels and plaques in CTA and TOF-MRA are magnified after 3D reconstruction, especially the changes in the position and size of the plaques would confuse clinical judgment.

### 3D Reconstruction of Vascular Lumen for Computational Fluid Dynamics

**Figure 3:**
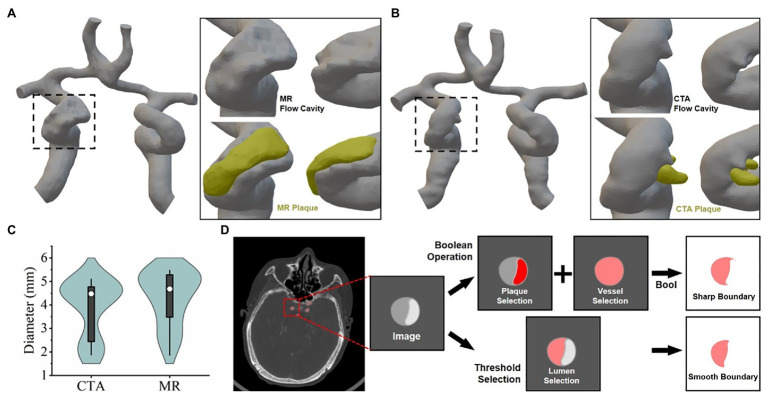
3D reconstruction of vascular lumen for computational fluid dynamics **(A)** and **(B)** respectively show the vascular flow lumen (left CTA, right TOF-MRA) reconstructed by the threshold screening method. **(C)** The frequency of vessel lumen with different diameters in the model schematic diagram. **(D)** Schematic diagram: the difference between the principle of image segmentation and the results by Boolean operation and threshold filtering method.

To further analyze and mine CTA and TOF-MRA images, computer fluid dynamics (CFD) is often used. This also means that the 3D reconstruction method described above would be executed, and more post-processing methods will be used. Firstly, we used the threshold box selection method instead of the Boolean algorithm (Figure 3D) to segment the blood vessels and then performed 3D reconstruction, which could reduce the sharpness of the boundary and obtain a smoother model surface. After the model of the flow cavity was obtained, the model of the plaque was segmented by the same method, and it was assembled in the corresponding position of the flow cavity of the blood vessel to facilitate comparison and observation. The results showed (Figures 3A,B) that the flow cavities and plaques of the two models could fit together and present different morphological features. As shown in the figure where the patch was located (black dashed rectangle), the flow cavity morphology of the two models was obviously different, and the location of the patch and the degree of indentation was also inconsistent. The 3D models reconstructed from CTA and TOF-MRA images had significantly different distribution rates for different lumen diameters (Figure 3C), which to some extent suggests their influence in subsequent fluid mechanics simulations. To some extent, this distribution suggests their influence in the subsequent fluid mechanic’s simulation.

### Fluid Dynamics Simulation Analysis *via* CTA and MR Imaging

**Figure 4:**
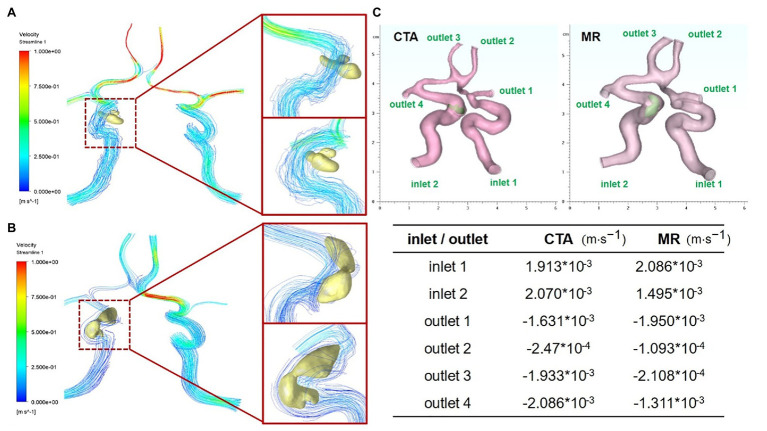
Fluid dynamic simulation *via* CTA and TOF-MRA imaging **(A)** Carotid artery and plaque flow field simulation *via* CTA imaging **(B)** carotid artery and plaque flow field simulation *via* TOF-MRA imaging. **(C)** Flow distribution difference at entrance and outlet of two models corresponding to CTA and TOF-MRA.

In order to further explore the difference in the effect of carotid artery stenosis on blood flow under CTA and TOF-MRA imaging, the finite element analysis method of fluid mechanics was used for analysis. We firstly obtained the CTA and TOF-MRA flow cavity models of the patient at the same lesion, used the finite element simulation software Ansys’ Fluent module to perform fluid dynamics simulation analysis on the two models (Figures 4A,B), and then counted the distribution difference of inlet and outlet flow rates. The results show that due to the slower blood flow velocity at the entrance (1.913 × 10^−3^ m/s, 2.070 × 10^−3^ m/s), the flow line in the flow cavity near the entrance was relatively stable, while the turbulent flow in the narrow cavity with plaques was increased, and finally, the flow velocity was greater near the outlet with a smaller diameter. The flow cavity obtained by TOF-MRA imaging was narrower with a more unstable streamline, which further leads to a difference in the distribution of flow output after plaque stenosis (Figure 4C).

## Discussion

Early detection and correct evaluation of vascular wall atherosclerotic plaques play an important role in clinical prevention and treatment. Although many scholars believe that DSA is the “gold standard” for the diagnosis of carotid artery disease, it is traumatic and may cause serious complications, such as vasospasm and plaque shedding, and it is often difficult for patients with mild symptoms to accept this examination and the DSA examination. Therefore, more and more attention has been paid to CTA and MR imaging of the carotid artery considering their easy operability with high image resolution (De Weert et al., 2009; Gupta et al., 2015b). In this research, we imported 3D original image data from CTA and TOF-MRA through MIMICS software and modified the final output of the 3D plaque model without any image modification. We compared the model accuracy of carotid plaque with CTA and TOF-MRA and found our method greatly improved the operational simplicity, automatization, and stability.

Using MIMICS software to perform 3D automatic segmentation directly is very simple and time-saving, which only requires us to do little preparatory work, that is, to provide the original image in DICOM format. The average time of automatic segmentation of CTA and MRA images into standard template files for 3D models using MIMICS software is 20 min. At the same time, we consulted two different physicians with rich experience in imaging diagnosis to help assess manual segmentation and avoid personal subjective errors. The future development of angiography technology and equipment will further improve the image quality of CTA and MRA, which will definitely simplify and improve the accuracy of plaque segmentation.

Improving image quality is helpful to identify carotid atherosclerotic plaque risk factors (Abbott and Nicolaides, 2015). CTA has been widely used in the field of vascular imaging in recent years, and MR has been proposed to replace CTA (Zhao et al., 2007; Qiao et al., 2012) because of its advantages in soft tissue detection. In this research, we had implemented two-dimensional image segmentation, 3D reconstruction of blood vessels and plaques, 3D reconstruction of the vascular lumen, and a hemodynamic simulation to compare the imaging features and post-processing potential between CTA and MR. The results showed that both CTA and MR could judge vascular plaque in clinical diagnosis, but CTA showed advantages when post-processing the image.

CTA imaging has high spatial resolution and could show the degree of lumen stenosis more clearly (Trelles et al., 2013). It has high sensitivity and specificity for luminal stenosis caused by carotid atherosclerotic plaque, the detection accuracy of small punctate calcified plaques is significantly better than MR particularly. The possible reason for the difference between CTA and MR imaging might be the difference in the components of the calcified plaques. The influence of this component difference on the MR imaging signal and the interference of surrounding tissue signals affects the judgment of the plaque boundary and ultimately leads to a large difference after the plaques are segmented and reconstructed.

In the current carotid plaque examination method, both CTA and MR can judge the degree of lumen stenosis. But the effect of CTA on the carotid atherosclerotic plaque was better than MR. MR overestimation of lumen stenosis will directly lead to large differences between MR and CTA diagnosis. Recently, enhanced MRA detection methods, such as Phase Contrast MRA (PC MRA), Time of Flight, TOF-MRA, and Dynamic Contrast Enhancement MRA (CE-MRA), can improve the spatial resolution of imaging. Reducing the effect of blood flow on plaque imaging can improve the accuracy of carotid atherosclerotic plaque diagnosis.

CTA and MR images show great differences after 3D reconstruction, especially the location and size of patches. This suggests that there are still shortcomings in the clinical application of image post-processing technology. The subtle optical density distribution differences resulting from these two imaging techniques will be amplified after multilevel image processing. Without the experience of clinicians, it is still difficult to interpret pathology independently by using only one imaging technique to display physiological details through image post-processing. Perhaps higher resolution or a combination of imaging techniques is still essential.

The threshold filtering and Boolean operation algorithm are usually used to segment the different components in grayscale images, which is usually not strictly stipulated. But when the segmented image is used for the later computational simulation, different segmentation methods, and segmentation order will result in different shapes, as shown in (Figure 3D). This has little effect on the CTA image because its gray gradient is easy to distinguish between the vascular cavity and the with the help of a contrast agent. However, the gray gradient of the MR image is relatively slow, and the optical density contrast between different materials is small. Multiple threshold box selection will cause serious distortion of the figure. The boolean algorithm is easy to produce sharp boundaries or isolated closed lumen. and the use of contrast agents can significantly improve the difficulty of image segmentation, especially when the image will be used for 3D reconstruction and mechanical simulation.

In summary, this study utilized a semi-automatic approach to evaluate the accuracy of carotid plaque models built from CTA and TOF-MRA source data. We compared two-dimensional images and three-dimensional models of carotid plaque obtained by two angiographic techniques. This research evaluated the potential of CTA and TOF-MRA imaging methods in the clinical diagnosis and fluid dynamics of carotid plaque and revealed the selectivity of image post-processing analysis for original medical image acquisition.

## Data Availability Statement

The raw data supporting the conclusions of this article will be made available by the authors, without undue reservation.

## Ethics Statement

The studies involving human participants were reviewed and approved by Ethics Committee of Chongqing People’s Hospital. Written informed consent to participate in this study was provided by the participants. Written informed consent was obtained from the individuals for the publication of any potentially identifiable images or data included in this article.

## Author Contributions

FD, KL, and MG conceived the article and were responsible for this research. LY and CM contributed to the original data acquisition, data curation, and data extraction. KL and MG: conceptualization and supervision. FD: model reconstruction and investigation. FD and RY: methodology. FD and MG: software. FD and CM: writing – original draft and writing – review and editing. All authors contributed to the article and approved the submitted version.

### Conflict of Interest

The authors declare that the research was conducted in the absence of any commercial or financial relationships that could be construed as a potential conflict of interest.
